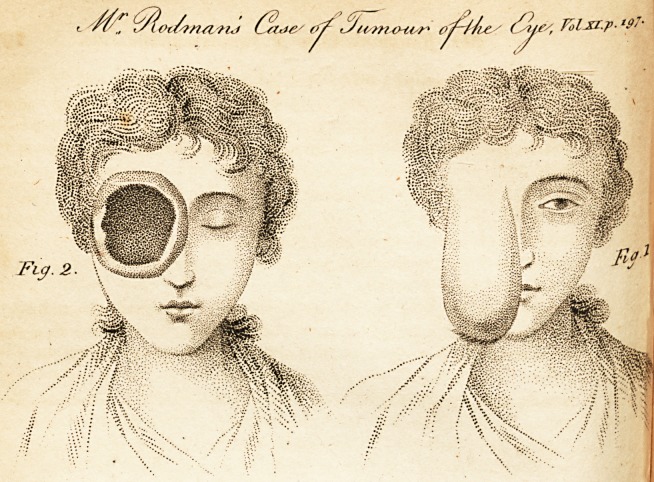# Case of Tumour of the Eye

**Published:** 1804-03-01

**Authors:** John Rodman

**Affiliations:** Paisley


Mr. Rodman's Case of Tumour of the Eye, Vol XI. p. 197.

### CASE OF TUMOUR OF THE EYE; *communicated by* MR. JOHN RODMAN, *of Paisley.*

[ With an Engraving. ]

IN August, 1801, Margaret M'Lean, ætat. 12, while amusing herself with some companions, accidentally struck her forehead against a wall, by which stroke she was at first a little stunned; but soon recovered, and resumed her play. No complaint was noticed till the fifth day after, when she felt a pain in the forehead, over the left eye. This pain increasing, inflammation and evident enlargement of the eye quickly succeeded, which being treated with leeches, blisters, eye-waters, &c. without the desired effect, she was admitted into the Glasgow Infirmary on the 7th of November following.

It appears by the first report, that she had a fixed pain in the left eye and forehead; the vessels of the adnata were universally red and turgid; the eye considerably protruded, forming a regular smooth tumour, except at one part, where a small portion of it extended beyond the palpebræ, and the sight was entirely gone. Her appetite was good, and the general state of her health little impaired. Common cataplasms were applied to the enlarged eye; the vessels of the adnata were divided; the cornea was opened with a lancet, and a small quantity of pus discharged, mixed with part of the aqueous humour. No material benefit having been derived from this variety of treatment, the surgeon in attendance proposed to extirpate the eye; but as she and her parents were averse to it, she was dismissed by desire on the 28th of the same month.

 Believing

Believing herself relieved by the use of bread poultices and rejecting every necessary medical advice, she persisted in applying them to the increasing tumour till the 18th of August, 1802, when, for the first time, I was desired to visit her, and took the following statement. Pulse 130, remarkably feeble; face and lips almost without a tinge of blood; her body is so emaciated, that it resembles a skeleton covered with delicate skin, through which the livid veins are seen. Notwithstanding the considerable quantities of laudanum given, to alleviate her pain and procure rest, she has not been known to enjoy sleep for several months. The tumour hangs down beyond the chin, and covers great part of the mouth; it is 7 ½ inches long; and being of a conical form, its base is 11 inches in circumference. The palpebræ are so very much extended as wholly to surround it. Fig. 1, represents this state of the tumour, with the cilia at the bottom. There is a foul ulcer between the cilia, from which frequent alarming hæmorrhagies have taken place since April last; another ulcer, formed a few days ago on the left side of the tumour, is spreading rapidly, and discharging such fœtid matter, that to remain in the room where she is, for a short time, becomes extremely distressing.

I was now told she had determined to have the tumour removed, and intreated to lose no time in preparing for the operation. To this I could by no means assent at once, having made the above observations; it was reasonable to believe from the general appearances that she could not survive many days; and should she die soon after the operation, I might not only be blamed for rashness, but it might be an instance ready to produce against a similar operation under different circumstances. With a view to satisfy her, however, I procured a consultation of my medical brethren, whose kind and useful assistance on such occasions, I take this opportunity gratefully to acknowledge; and after maturely deliberating upon the state of the patient, it was unanimously agreed to form some plausible apology for deferring the operation. I did so, but she received it with visible marks of disappointment. Early next morning I was sent for again; found her uncommonly anxious to have the operation performed, and, in the eagerness of desire, she wept, crying out, "If I recover, it will be unexpected happiness; I would die rather than exist in this state of pain and wretchedness." Accordingly, with proper assistance, I extirpated the tumour, removed all the diseased substance within the orbit, covered the cut surface with spunge to prevent any hæmorrhagygy, for she was very faint though she lost little blood, applied the usual dressings, and laid her to rest. For this state of it, see Fig. 2.

She slept well the first night, was much easier next day, seemed cheerful, and after expressing her thankfulness for what I had done, assured me that she found more ease since yesterday, than she experienced many months before. A serous bloody fluid oozed from the wound till the fourth day, when the dressings were removed; and as the spunge could not be taken away without giving great pain, I contented myself with cutting off a part of it at every dressing. This gave her no uneasiness, and the wound was cleared of it in six weeks. By that time the orbit was nearly filled with healthy granulations, and in every other respect she was doing well, suffering no pain, enjoying sound sleep, and taking food; in short, her health was so much restored, that she could attend to some amusements, and was frequently carried into the garden. Towards the end of the seventh week, a small livid fungous tumour appeared at the internal angle of the orbit, where a deficiency of bone was discovered during the operation. It resisted every variety of caustic applied, became firmer, and pushed the granulations aside. Ten days after the appearance of this tumour, the surface of the sore changed for the worse; her pulse quickened, with diminished appetite; the sight of the right eye gradually failed, till she became blind; diarrhœa ensued, ending in bloody stools; and she died in the eleventh week after the operation. Being sensible of approaching dissolution, the day before death, she remarked, with a considerable degree of gratitude, that "she suffered more pain in one day before the operation than she did ever since."

*Dissection, twenty-four hours after Death.*

Having exposed the contents of the cranium, no mark of disease could be discovered; but upon pressing behind the diseased orbit, a large hard body was felt. After cutting up the dura mater and removing the brain, which seemed free from disease, this body was found lying between the orbit and dura mater, to which it adhered by means of slender vascular filaments. It was of a white colour, very irregular, extending as far back as the sella turcica, and considerably to the right of the crista galli. Its surface was hard like a body approaching to ossification, but towards the base of the cranium it had a gelatinous appearance whiter, and more consistent than pus. The livid tumour observed on the seventh week after the operation which I attempted to destroy by caustics, was a branch from the internal tumour. Another branch had forced itself into the left nostril some time before death, which in every respect resembled a very firm polypus. On the left side there did not remain the smallest vestige of the optic, nor any nerve to the seventh. The right optic nerve was preternaturally enlarged and surrounded by the internal tumour, from the sella turcica forward. After all the diseased substance was extracted, a most extraordinary deficiency of bone was observed. The left orbitar processes of the frontal, malar, and maxillary bones were consumed, except at the external canthus, where a small portion of the two first bones remained, carious, and thickened. The os unguis, with the whole left side of the sphenoid and ethmoid bones were wanting; indeed, every portion of bone which had been in contact with the tumour was either softened or destroyed. A probe passed freely through the body of the sphenoid bone into the nostrils and palate. Another tumour, which had appeared for some weeks, extended before death from above the superciliary ridge to the apex of the nose; it was an inch and a half broad, and near an inch deep. The bones with which it was connected were also softened, and though it resembled the firmest part of the internal tumour in colour and consistence, they were totally unconnected.

*December* 19, 1804.

## Figures and Tables

**Fig. 1 Fig. 2 f1:**